# Evidence of infectivity of airborne porcine epidemic diarrhea virus and detection of airborne viral RNA at long distances from infected herds

**DOI:** 10.1186/s13567-014-0073-z

**Published:** 2014-07-14

**Authors:** Carmen Alonso, Dane P Goede, Robert B Morrison, Peter R Davies, Albert Rovira, Douglas G Marthaler, Montserrat Torremorell

**Affiliations:** 1 Department of Veterinary Population Medicine, College of Veterinary Medicine, University of Minnesota, Saint Paul, Minnesota, USA

## Abstract

Porcine epidemic diarrhea virus (PEDV) spread rapidly after being diagnosed in the USA in April 2013. In this study we assessed whether PEDV could become airborne and if so, whether the virus was infectious. Air samples were collected both from a room containing experimentally infected pigs and at various distances from the outside of swine farms experiencing acute PEDV outbreaks. Results indicated presence of infectious PEDV in the air from experimentally infected pigs and genetic material of PEDV was detected up to 10 miles downwind from naturally infected farms. Airborne transmission should be considered as a potential route for PEDV dissemination.

## Introduction, methods and results

Porcine epidemic diarrhea virus (PEDV) is an enveloped, single-stranded positive sense RNA virus that belongs to the *Coronaviridae* family, subfamily *Coronavirinae*, genus *Alphacoronavirus* [[1]]*.* PEDV was first observed in feeder pigs and fattening swine in England and identified and named as the main causative agent of porcine epidemic diarrhea (PED) in 1978 [[2],[3]]. The main clinical sign of PEDV is watery diarrhea with 100% morbidity and 50-100% mortality in piglets up to 1 week of age, and less severe disease in older pigs including sows. Epidemic outbreaks of PEDV have been reported from different countries in Europe as well as Asia, including Japan, China, South Korea and Thailand [[4]–[7]]. Since late 2010, a remarkable increase in PEDV outbreaks has been reported in pig-producing provinces in China resulting in tremendous economic losses attributed to the emergence of new strains [[8]].

PEDV was first diagnosed in the eastern Midwest region of the United States in April 2013 [[9]]. Rapid spread of PEDV was evidenced by the diagnostic submissions to specialized diagnostic laboratories. At 4 and 8 weeks after the first case was diagnosed, the number of states with positive cases increased from 6 to 12. At that time, Iowa and Oklahoma were the states with the most diagnostic case submissions testing positive for PEDV [[10]]. Subsequently, PEDV has continued to spread rapidly and as of June 2014 has been confirmed in 30 states in the USA [[10]].

Although PEDV infection among pigs occurs predominantly by the fecal-oral route, airborne transmission of PEDV among farms has been suspected due to the rapid regional spread of PEDV, but remains unproven. Airborne transmission of bacteria and viruses originating from aerosolized particles from defecating and vomiting individuals has been previously reported [[11],[12]]. Furthermore, farms located in close proximity to a known infected farm have increased odds of being PEDV infected. Recently, detection of PEDV RNA attributed to airborne spread within a space housing experimentally infected pigs was reported [[13]]. However, because sentinel pigs did not succumb to PEDV infection, airborne transmission was not confirmed. Based on the hypothesis that PEDV particles can be suspended in the air and remain infectious while airborne, our objective was to determine whether infectious PEDV could be detected in air samples under both experimental and field conditions.

To evaluate whether PEDV could become airborne and remain infectious in air samples, we performed an experimental challenge study. Briefly, twelve 7 to 8 week-old PEDV-naïve pigs were housed in a 35 m^3^ BSL-2 animal isolation unit at the University of Minnesota. Prior to inoculation pigs were tested by RT-PCR to confirm their PEDV negative status. On day 0 of the study, each pig was intragastrically inoculated with 20 mL of PEDV inoculum calculated from preliminary pilot studies as an adequate volume to properly deliver the samples without over-filling the stomach. The inoculum was derived from mucosal scrapings of a PEDV case submitted to the University of Minnesota Veterinary Diagnostic Laboratory (UMVDL). The inoculation material was confirmed positive by PEDV RT-PCR and diluted to an estimated number of RNA copies/mL, based on a standard curve, ranging from 3.96 × 10^10^ to 7.57 × 10^10^ (corresponding to values of 15 to 16 cycle threshold or Ct). The inoculation material was prepared and kept refrigerated for 24 h at 4 °C prior to inoculation. After inoculation, clinical signs of PEDV infection including body temperature, diarrhea, vomiting and lethargy were recorded for 3 days at each sampling event from 24 h post challenge. Air samples were collected by placing a cyclonic air collector (Midwest Micro-Tek, Brookings, SD, USA) in the center of the isolation room 1.2 m above the floor. Pigs did not have direct access to the air sampler. Samples were collected for 30 min into 10 mL of viral growth media (minimum essential media (MEM) supplemented with 3% fetal calf serum) placed in the air cyclonic collection vessel as previously described [[14]]. Samples were collected twice daily (morning and afternoon) and two air samplers were used between sampling events to enable effective disinfection of the devices. Pre-collection negative controls were taken by swabbing the collector vessel and spinning mechanism using a sterile swab with Stuart’s medium (BBL CultureSwab™, Dickinson and Com., Sparks, Maryland, USA). After collection, samples were stored at −80 °C until the termination of the study when all samples were submitted to the University of Minnesota Veterinary Diagnostic Laboratory (UMN VDL) for PCR analysis.

To determine whether PEDV could become airborne under field conditions, 8 swine herds located in Oklahoma (US) and belonging to one production company were identified. Finishing and breeding herds that were experiencing acute PEDV outbreaks during each day of air collection throughout the study were purposively selected. Air collection was conveniently performed at different locations downwind, ranging from 0.006 to 15 miles (9.6 m to 24.14 km) from each of the farms starting from the furthest distances each day. A total of 62 samples were collected using an air cyclonic collector as described above for 30 min, with samples stored on dry ice and submitted to the UMN VDL for PEDV RT-PCR testing.

To assess the infectivity of the air samples, a bioassay consisting of inoculating susceptible piglets with the air samples was performed. The sensitivity of the bioassay protocol was previously demonstrated with an infectious dose serial dilution pilot study tested in piglets of the same age and number of piglets per sample used in this study (unpublished data). Briefly, 10-day-old pigs from a PEDV-negative farm were purchased and each pig allocated to a separate isolation room. On arrival pigs were rectal swabbed and confirmed negative by PEDV RT-PCR. Inoculation material per pig consisted of a 2 mL of a single sample (field samples) or a 2 mL pool from 3 samples (experimental samples) diluted 1:10 with phosphate-buffered saline (PBS) to obtain a total of 20 mL of inoculation material per pig. Pigs were inoculated after 1 day of acclimatization via gavage using a Kendall 16 inch #14 French nasoesophageal tube placed orally into the esophagus. Entry of the tube into the stomach was confirmed by negative pressure with a 20 mL syringe on the esophageal tube. Control samples included a sample collected from a PEDV confirmed positive pig with a Ct value of 16 (positive control), one sample diluted 10^−7^ times from the positive control (intended Ct 36–38), and one negative control sample consisting of sterile PBS. Clinical signs of diarrhea, vomiting, and dehydration were monitored every 12 h throughout the bioassay. All pigs were euthanized 48 h post-exposure by injection of 2 mL of pentobarbital (Fatal-Plus®, 100 mg/kg IV) into the external jugular vein. Intestines were removed immediately and sections of jejunum and ileum from 6 different areas were collected for histopathological and PCR analysis. Both tissue and air samples were tested by real time RT-PCR. Briefly, RNA was extracted from intestinal and environmental samples using the MagMAX™-96 Viral RNA Isolation Kit (Applied Biosystems, Foster City, CA, USA), according to the manufacturer’s instructions. Molecular detection of PEDV by quantitative RT-PCR (RT-qPCR) used spike gene primers (Forward 1910: ACGTCCCTTTACTTTCAATTCACA and Reverse 2012: TATACTTGG TACACACATCCAGAGTCA) and probe (1939: FAM-TGAGTTGATTACTGGCACGCCTAA ACCAC-BHQ). The primer and hydrolysis probe set utilized the AgPath-ID™ One-Step RT-PCR Reagents (Life Technologies, Grand Island, NY, USA) and 5 μL of RNA with the 7500 Fast Real-Time PCR System (Life Technologies, Grand Island, NY) on the Fast Mode setting under the following thermal cycling conditions, reverse transcription, 10 min at 48 °C; Taq activation, 10 min at 95 °C; followed by 40 cycles of 15 s at 95 °C and 45 s at 60 °C. A gBlocks Gene Fragments (IDT) containing the PEDV gene target was ordered and a 10 fold serial dilution was run on the PEDV RT-qPCR generating a standard curve with a slope of y = 9E = 12e^-0.648x^, which was used to transform the Ct values into estimated copies of PEDV RNA per mL of original material.

Procedures described in this study followed approved protocols by the University of Minnesota Institutional Animal Care and Use Committee protocol number 1110A5802, and Institutional Biosafety Committee protocol number 1208H18341.

**Figure 1:**
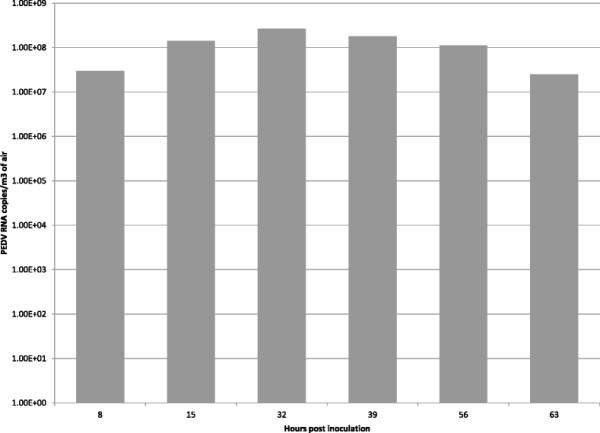
**PEDV RT-PCR results from air samples collected from experimentally infected pigs.** Total PEDV RT-PCR positive results from air samples collected during the experimental. Results are reported as estimated PEDV RNA copies per m^3^ of air and hours post-inoculation.

PEDV was demonstrated in all air samples collected from the experimentally infected pigs (Figure 1). The estimated number of RNA copies per m^3^ of air ranged between 1 × 10^6^ to 1 × 10^9^, and Ct values ranged from 26.3 to 22.7. Negative control samples tested negative. Pigs infected with experimental air samples experienced moderate to severe diarrhea, with fecal number of RNA copies/mL ranging from 3.96 × 10^10^ to 7.57 × 10^10^ (Ct values ranged from 15 to 16). These pigs had histopathological lesions of moderate to marked atrophic enteritis. Pigs from the negative control group showed no clinical signs, tested negative by PCR and had normal intestinal histomorphology.

Eleven of 62 (18%) air samples collected under field conditions tested RT-PCR positive, with number of PEDV RNA copies/m^3^ of air sampled ranging from 4.99 × 10^5^ (at 0.01) to 4.21 × 10^3^ (at 1 mile) (Table 1). Genetic material of PEDV (7.98 × 10^3^ PEDV RNA copies/m^3^) was detected up to 10 miles downwind of farm C. At least one air sample from each farm (except farms E and H) tested positive. The mean number of PEDV RNA copies/m^3^ of air for samples collected under field conditions (9.64 × 10^4^ ± 1.5 × 10^5^) was lower (Student *t* test, *p* < 0.001) than that of samples collected experimentally (1.25 × 10^8^ ± 9.16 × 10^7^). None of the air samples collected under field conditions were positive on bioassay.

**Table 1:** **Results from PEDV RT-PCR positive air samples (reported as PEDV RNA copies/m**^**3**^**of air) collected under field conditions relative to sampling location and proximity to the farm**

**Farm identification**	**Type of farm (Breeding or Finishing)**	**Distance from farm (miles)**	**RT-PCR PEDV (RNA copies/m**^**3**^**of air)**
A	Breeding	0.01	2.05 × 10^5^
B	Breeding	0.01	1.81 × 10^5^
G	Finishing	0.01	4.99 × 10^5^
A	Breeding	0.50	4.04 × 10^4^
C	Breeding	1.00	2.65 × 10^4^
C	Breeding	1.00	2.07 × 10^4^
G	Finishing	1.00	4.21 × 10^3^
C	Breeding	3.00	1.73 × 10^4^
F	Finishing	3.00	3.50 × 10^4^
G	Finishing	3.00	2.36 × 10^4^
C	Breeding	10.00	7.98 × 10^3^

## Discussion

PEDV was considered exotic to the North American swine population until it was first diagnosed on April 2013. Once PEDV diagnosis was confirmed the infection spread very rapidly among swine herds causing devastating losses to producers. Because of the rapid spread of the virus among farms, aerosol transmission was suspected in some areas where virus transmission could not be explained by any other known routes. In our study, we assessed the detection and viability of PEDV in air samples collected from a room housing experimentally infected PEDV pigs. Pigs inoculated with diluted air samples developed PED clinical signs and lesions and PEDV was detected in feces and tissues indicating that air samples contained infectious PEDV. Secondly, we investigated the transport of PEDV under field conditions and we showed that PEDV genetic material could be detected up to 10 miles although infectivity could not be shown. To the authors’ knowledge, this is the first report to establish that PEDV can be found in the air, that suspended airborne particles can be infectious, and that PEDV genetic material can be transported over long distances.

Evidence of airborne transmission of enteric pathogens (bacteria and viruses) has been shown previously [[12]]. Drying of swine feces due to increase in temperature and resuspension of feed deposited on the floor of pig buildings can result in the generation of dust able to adsorb and carry microorganisms and odorous compounds [[11]]. We speculate that this is one likely mechanism by which PEDV became aerosolized and remained suspended in the air, and may contribute to both within farm transmission and area spread in high density pig regions. Furthermore, PEDV has a low infectivity dose and is able to survive in the environment for extended periods facilitating the risk of disease transmission [[15]].

Bioassay completed with air samples collected under experimental conditions demonstrated that PEDV could remain infectious while airborne. However, inoculation with samples collected under field conditions did not result in PEDV infection. The lack of infectivity could be attributed to the lower viral concentration in the field samples or the inactivation of the virus by temperature, solar light intensity, ultraviolet radiation, or during sample storage. Field samples were collected during the month of July in the Oklahoma panhandle region at a time of high environmental temperature (ranging from 15 to 28.9 °C) and low relative humidity (ranging from 23 to 67%). Further research is needed to determine the airborne infectious dose needed to induce PEDV infection and the impact of environmental factors such as UV light, relative humidity and temperature on PEDV viability.

In summary, we have demonstrated that PEDV can become airborne, remain infectious while suspended in the air, and that PEDV genetic material can be transported long distances following natural infections. Further studies are needed to assess the risk of airborne transmission of PEDV among farms and the need to address this route in biosecurity and health control programs.

## Competing interests

The authors declare they have no competing interests.

## Authors’ contributions

CA helped to design and performed the animal experiment and wrote the manuscript. MT designed the experimental study and helped draft the manuscript. DG helped to design and performed the field sampling and bioassay experiments and helped draft the manuscript. PD, RM and AR contributed to the elaboration of the study design, interpretation of the results and helped draft the manuscript. DM developed the diagnostic procedures and helped with the manuscript. All authors read and approved the final manuscript.
